# Reversible Right-Sided Heart Failure Secondary to Carcinoid Crisis

**DOI:** 10.1155/2013/487801

**Published:** 2013-10-01

**Authors:** Mariana Soto Herrera, José A. Restrepo, Jesús H. Díaz, Andrés Ramos, Andrés Felipe Buitrago, Mabel Gómez Mejía

**Affiliations:** ^1^Critical Care Department, Hospital Universitario Fundación Santa Fe de Bogotá, Calle 119 No. 7–75, Bogotá 110111, Colombia; ^2^Universidad de los Andes, Cra 1 No. 18A-12, Bogotá 111711, Colombia; ^3^Internal Medicine Department, Cardiology Section, Hospital Universitario Fundación Santa Fe de Bogotá, Calle 119 No. 7–75, Bogotá 110111, Colombia

## Abstract

Carcinoid crisis is an infrequent and little-described complication of neuroendocrine tumors that can be life threatening. It may develop during induction of anesthesia, intraoperatively, during tumor manipulation and arterial embolization, or even spontaneously. The massive release of neuroendocrine substances can lead to potentially fatal complications. Somatostatin analogs inhibit the release of these substances and are the mainstay of treatment. The following case report describes a patient with reversible acute right-sided heart failure posterior to hepatic artery embolization.

## 1. Case Presentation

**Figure 1:**
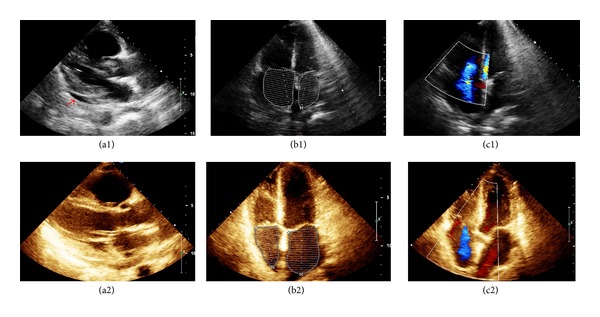
Transthoracic echocardiogram. (a1) Long parasternal axis showing pericardial effusion (arrow); (a2) one week later showing resolution of pericardial effusion; (b1) four-chamber view showing marked right atrial dilatation; (b2) four-chamber view one week later showing normalization of right atrial size; (c1) color doppler showing moderate tricuspid regurgitation; (c2) color doppler one week later showing a decrease in tricuspid regurgitation.

A 41-year-old female patient without a significant medical history presented symptoms that evolved over a period of one year, initiating with jaundice and epigastric pain. A computerized axial tomography of the abdomen was performed, which showed an increased liver size with multiple hepatic lesions and dilation of the intrahepatic bile duct. Magnetic resonance cholangiography evidenced a solid 9 cm mass and lesions suggestive of hepatic neoplastic compromise. A hepatic biopsy was done, which documented low grade compromise caused by a neuroendocrine tumor (grade 1). The OctreoScan showed multiple enhancing lesions in the liver with multifocal tumoral compromises with somatostatin receptors. Positron emission tomography evidenced hypermetabolic hepatic lesions consistent with a malignant pathology. Initial workup showed chromogranin A, 99.5 *μ*g/L, and 5-hydroxyindoleacetic acid, 10.3 mg. A diagnosis of low grade neuroendocrine tumor (well-differentiated tumor, histological grade WHO 2), with unknown primary and with documented hepatic metastases, was made. The case was presented to the neuroendocrine tumors committee and treatment with transarterial hepatic embolization was considered. A preprocedural transthoracic echocardiogram was conducted, which appeared normal. Initially, the patient was in a good general state. Blood pressure was 130/70 mmHg, and heart rate was 75 beats/minute, without other significant findings on physical examination. On the second day of hospitalization, hepatic arterial embolization was performed without chemotherapy using polyvinyl alcohol particles, without complications. On the fourth day of hospitalization, the patient presented deterioration in the functional class and on physical examination presented moderate jugular ingurgitation and edema of lower limbs. Computerized axial tomography was performed, which ruled out pulmonary thromboembolism; a transthoracic echocardiogram showed left ventricular systolic function in the lower normal limit (ejection fraction 51%), moderate dilation of right-sided chambers with decreased systolic function, and moderate tricuspid regurgitation (Figure 1). A right-sided heart failure secondary to carcinoid crisis was considered; the patient was treated with octreotide 100 *μ*g/hour in continuous infusion and furosemide and was transferred to the intermediate care unit. Control chromogranin A levels were 652.10 *μ*g/L. The patient's clinical condition progressed, with an improvement in her dyspnea, a decrease in the edema of lower limbs, and resolution of the jugular ingurgitation and hepatojugular reflux. Octreotide infusion was reduced to 70 *μ*g/hour. However, the patient again presented mild jugular ingurgitation and positive hepatojugular reflux and moderate lower limbs edema. Chromogranin A levels were 488.40 *μ*g/L. Octreotide infusion was increased to 100 *μ*g/hour. A new transthoracic echocardiogram evidenced biventricular systolic function within normal parameters with moderate tricuspid regurgitation (Figure 1). An aldosterone antagonist and a beta blocker were started. The patient's overall clinical condition improved, tolerating a progressive reduction of octreotide infusion subsequently replaced with subcutaneous octreotide. Monitoring of chromogranin A levels showed a decline to 302.80 *μ*g/L and the patient was discharged. 

## 2. Discussion

Carcinoid tumors are neuroendocrine malignancies that generally originate in the enterochromaffin cells of the gastrointestinal tract [1]. They affect up to 2.1 per 1,000 people of the general population [2]. They are characterized by insidious growth that produces few or no symptoms until they have reached great size or developed distant metastasis, generally in the liver. Tumors localized in the midgut usually synthesize, store, and release vasoactive substances including 5-hydroxytryptamine, tachykinins, histamine, and prostaglandins [1, 3, 4]. The term “carcinoid syndrome” describes complex clinical manifestations produced by the chronic liberation of one or more of these substances. Systemic manifestations vary depending on the embryological origin of the primary tumor and the extension or site of metastasis [5, 6]. This occurs in approximately 20 to 30% of patients with carcinoid tumors with hepatic metastasis [7]. 

Carcinoid crisis is an infrequent and seldom described complication of neuroendocrine tumors that can be life threatening; most are secondary to chronic cases of carcinoid syndrome. Carcinoid crisis may develop during induction of anesthesia, intra-operatively, during tumor manipulation and arterial embolization, or even spontaneously [1, 2]. The massive liberation of neuroendocrine substances can lead to complications, some of which are potentially fatal, such as flushing, severe hypotension, paroxysmal hypertension, bronchial spasms, edema, cardiac arrhythmias, metabolic acidosis, and altered state of consciousness [6]. Cases of hypotension are usually difficult to treat because they are resistant to sympathomimetics. These agents can also induce further liberation of vasoactive substances by the tumor [8]. There are documented cases of carcinoid crisis presenting as right-sided heart failure [4, 6, 9]. Somatostatin analogs, such as octreotide, inhibit the liberation of these neurohumoral substances by the tumor and are the mainstay of treatment [1, 2]. Octreotide intravenous infusion (50–100 *μ*g/hour) has been described and should be administered approximately two hours before initiating the procedure; the infusion should continue for 48 hours after the procedure. These patients might require continuation of treatment in order to manage carcinoid syndrome [1]. 

Carcinoid heart disease has been described in more than 50% of patients diagnosed with carcinoid syndrome. Cardiovascular effects are caused by the paraneoplastic effect of serotonin and other biogenic amines released by the carcinoid tumor, rather than direct metastatic involvement of the heart. These products are usually metabolized in the liver, but metastases may allow large quantities of these substances to reach the systemic circulatory system and the right side of the heart, without being inactivated [10]. Cardiovascular complications include structural lesions and hemodynamic alterations. Characteristic findings are plaque-like deposits of fibrous tissue at the endocardium that mainly affect the right side of the heart, causing thickening, retraction, and immobilization of the cusps of the tricuspid and pulmonary valves. The predominant consequence is the generation of tricuspid regurgitation and pulmonary stenosis. Structural alterations of the right-sided heart chambers can cause an increase in pressure, both in the atria and ventricle and may lead to right-sided heart failure. It is less common that the left side of the heart is affected because the substances are inactivated at the bronchial level. However, patients with bronchial tumors or patent foramen ovale may have left-sided chamber involvement [9]. 

Management of this pathology can be challenging and a multidisciplinary approach to patient care is advised [2]. An initial echocardiogram should be obtained in order to evaluate the presence of carcinoid heart disease. Patients presenting mild or moderate symptoms of right-sided heart failure can be treated using somatostatin analogs or diuretics and digoxin. Cases resistant to medical treatment should be considered for surgical valve replacement. However, this treatment can have high perioperative morbimortality rates [9]. 

This case represents a reversible right-sided heart failure following embolization of the hepatic artery. Symptoms were triggered by the liberation of vasoactive amines during the procedure. Management consisted in carcinoid crisis control with somatostatin analogs and symptom relief with diuretics to manage venous congestion, ultimately achieving reversal of heart failure. Preoperative octreotide was not used with this patient because the tumor was nonsecretory. Nevertheless, it could be appropriate to treat patients with nonproductive tumors with somatostatin analogs, given the risk of a carcinoid crisis occurring peri- or postprocedurally as a result of the liberation of vasoactive amines with tumor stimulation.

We decided to present this case because carcinoid crisis presented with only cardiovascular involvement, differentiating it from other previously described cases in which compromise included flushing, diarrhea, hypo- or hypertension, and/or bronchoconstriction. Additionally, we are aware of only just one published case presenting reversible right-sided heart failure secondary to carcinoid crisis.
